# Altered Metabolic Phenotype of Immune Cells in a Spontaneous Autoimmune Uveitis Model

**DOI:** 10.3389/fimmu.2021.601619

**Published:** 2021-07-27

**Authors:** Claudia Barfüßer, Carmen Wiedemann, Anne L. C. Hoffmann, Sieglinde Hirmer, Cornelia A. Deeg

**Affiliations:** Chair of Physiology, Department of Veterinary Sciences, Ludwig-Maximilians-Universität (LMU) Munich, Martinsried, Germany

**Keywords:** immunometabolism, PBMC, CD4^+^ T cell, OXPHOS, glycolysis, mitochondria, autoimmunity, equine recurrent uveitis (ERU)

## Abstract

As one of the leading causes of blindness worldwide, uveitis is an important disease. The exact pathogenesis of autoimmune uveitis is not entirely elucidated to date. Equine recurrent uveitis (ERU) represents the only spontaneous animal model for autoimmune uveitis in humans. As the metabolism of immune cells is an emerging field in research and gains more and more significance to take part in the pathogenesis of various diseases, we conducted experiments to investigate the metabolism of immune cells of ERU cases and healthy controls. To our knowledge, the link between a deviant immunometabolism and the pathogenesis of autoimmune uveitis was not investigated so far. We showed that PBMC of ERU cases had a more active metabolic phenotype in basal state by upregulating both the oxidative phosphorylation and the glycolytic pathway. We further revealed an increased compensatory glycolytic rate of PBMC and CD4^+^ T cells of ERU cases under mitochondrial stress conditions. These findings are in line with metabolic alterations of immune cells in other autoimmune diseases and basic research, where it was shown that activated immune cells have an increased need of energy and molecule demand for their effector function. We demonstrated a clear difference in the metabolic phenotypes of PBMC and, more specifically, CD4^+^ T cells of ERU cases and controls. These findings are another important step in understanding the pathogenesis of ERU and figuratively, human autoimmune uveitis.

## Introduction

Equine recurrent uveitis (ERU) is an important disease in horses worldwide (1, 2), which, being untreated, eventually leads to loss of vision in the affected eyes (3). It is characterized by recurrent and painful episodes of inflammation, which are mainly caused by autoaggressive CD4^+^ T cells that migrate from the peripheral blood through the blood retinal barrier into the eye to attack and destroy retinal structures (1). Few monocytes can be observed among these infiltrating CD4^+^ T cells, but the driving force during an uveitic attack are the CD4^+^ T cells (4, 5). Like in other autoimmune diseases, the pathogenesis of ERU is still not fully elucidated (1, 6). ERU represents the only spontaneous animal model for human autoimmune uveitis (HAU), a sight-threatening disease with a similar pathogenesis as ERU (1, 7–9). Thus, new findings concerning the underlying pathogenesis of ERU are of great value for both, veterinary and human medicine. For example, our group identified CRALBP as autoantigen in horses with ERU (10). Later on, CRALBP was also verified as important autoantigen in human autoimmune uveitis (11), autoimmune retinopathy (12) and juvenile idiopathic arthritis-associated uveitis (JIAU) (13). Patients with autoimmune uveitis receive long-term treatment with immune suppressants like high-dosed corticosteroids, cyclophosphamides or azathioprine or they are treated with antibodies against TNFα or IL-17 [reviewed in (14)]. Unlike these human patients, horses affected by ERU are only treated for a few days when they experience an acute inflammatory episode and not in the quiescent stage of the disease (5, 15, 16), therefore it is possible to examine immune reactions in the quiescent stage unaffected by long-term treatment.

The area of immune cell metabolism has gained more and more interest in the last decades. Immune cells produce ATP and other metabolites through metabolic pathways, which are closely connected with each other. The two major pathways for an immune cell to obtain ATP are oxygen-consuming oxidative phosphorylation (OXPHOS) and glycolysis, in which ATP is generated *via* oxygen independent substrate-level phosphorylation reactions [reviewed in (17, 18)]. Changes in metabolism are interdependent with oxidation-reduction (redox) reactions, for example electrons derived in glycolysis and the tricarboxylic acid (TCA) cycle are necessary to generate reducing equivalents contained in NADH. The NAD^+^/FAD coenzymes can be seen as the main oxidizing agents in cellular catabolism (19, 20). They are needed to transfer electrons from glucose (glycolysis), fatty acids (β-oxidation) and activated acetic acid (TCA cycle) to the mitochondrial respiratory chain for ATP generation [reviewed in (21)]. Most cells can quickly adapt to environmental changes and varying energy sources by switching between the dominantly used energy-delivering pathways (22). The interrelation between immune cell metabolism and the cell’s activation status was revealed through research in the last two decades (23). Naïve CD4^+^ T cells primarily rely on OXPHOS to generate ATP, for which the TCA cycle delivers electrons stored in NADH and FADH_2_ (21, 24). Upon activation, CD4^+^ T cells raise glycolysis not only for ATP production, but also to generate metabolic intermediates such as pyruvate, which are crucial for synthesizing amino acids and fatty acids to cover the demand for proliferation and effector function [reviewed in (21)]. Furthermore, different metabolic pathways dominate in CD4^+^ T cell subsets. For example, memory T cells rely primarily on catabolic pathways such as OXPHOS (including fatty acid oxidation as electron source) to cover their metabolic needs, while effector T cells (Th1, Th2 and Th17) exhibit a glycolysis-dominated metabolism (25, 26).

A first approach to identifying changes in metabolism of healthy and degenerated cells was taken over 60 years ago, as tumor cells were shown to shift from oxidative phosphorylation to the glycolytic pathway, more precisely to aerobic glycolysis (27–29). This showed that healthy and cancer cells, which have the same origin, can be distinguished upon their different metabolism. The microenvironment also affects cell metabolism, as it was shown in human T cell subsets in healthy and cancerous tissue (30). The CD4^+^ T cells in tumorous environment displayed decreased mitochondrial enzyme activity and reduced aerobic glycolysis compared to CD4^+^ T cells in an healthy microenvironment (30). Thus, cells with the same protein profile can be differentiated by their distinct metabolism in a healthy or tumorous microenvironment (30). Interestingly, the meaning behind the term “aerobic glycolysis” differs upon context. In cancerous cells, aerobic glycolysis, also known as the Warburg effect, describes their ability to convert pyruvate to lactate in the presence of oxygen (30, 31). In healthy cells, this reaction re-oxidizes NADH for continuing glycolysis under anaerobic conditions (32). Concerning biochemistry and activated immune cells, the term “aerobic glycolysis” mainly describes the conversion of pyruvate to Acetyl-CoA, which fuels the tricarboxylic acid cycle, in the presence of oxygen (27). To sum it up, cells in health and disease exert a distinct metabolic phenotype despite expressing the same protein profile.

Furthermore, metabolic aberrations of immune cells in autoimmune diseases are implicated to contribute to their autoaggressive and inflammatory phenotype (17). For example, systemic lupus erythematosus (SLE) CD4^+^ T cells of lupus-prone mice had enriched OXPHOS and glycolytic pathways and combined treatment with 2-DG and metformin *in vivo* could normalize metabolism of these CD4^+^ T cells and reverse biomarkers of the disease (33). This underlines the importance of not only investigating metabolic features of healthy immune cells, but to uncover possible differences in metabolism of immune cells of diseased individuals. However, over the last years, studies on spontaneous autoimmune uveitis mostly concentrated on different protein expressions in healthy and autoimmune cells. For example, our group previously demonstrated a correlation of higher integrin-linked kinase expression and a lower apoptosis rate of ERU lymphocytes, whereas necrosis rates were not significantly different, compared to cells of eye-healthy horses (6). To our knowledge, studies to distinguish autoaggressive immune cells from normal immune cells upon their metabolic phenotype were not yet performed in context of spontaneous autoimmune uveitis in humans or horses, although immunometabolism is considered to play a tremendous role in autoimmune diseases (29). Thus, the goal of the study was to characterize the metabolic features of equine PBMC and CD4^+^ T cells in health and ERU to detect possible alterations in the uveitic phenotype. This could contribute to identify and possibly even target the autoaggressive cells therapeutically in the peripheral blood before they migrate into the eye and attack retinal tissue.

## Materials and Methods

### Isolation of Primary Peripheral Blood Mononuclear Cells and Ethics Approval for Animal Research

Peripheral blood mononuclear cells (PBMC) of 25 healthy and 29 ERU cases were used in this study. Horses were regarded as eye-healthy, when no previous ocular inflammation was reported by the owner and the standard clinical routine examination did not indicate pathophysiological changes in the eye. These eye-healthy horses are referred to as controls in the following parts of the manuscript. Horses with ERU were diagnosed by experienced clinicians of the Equine Hospital of LMU Munich based on typical clinical signs of uveitis along with a documented history of multiple episodes of inflammation in the affected eye (34). All ERU blood samples were obtained in the quiescent stage of the disease. Equine whole blood samples of controls and ERU cases were taken from the *vena jugularis* in tubes with heparin sodium (50 I.U. per ml blood; Ratiopharm, Ulm, Germany). After sedimentation of blood components, the leukocyte-rich plasma was isolated by density gradient centrifugation (room temperature, 350 x g, 25 min, low acceleration, low break) using Pancoll separation solution (PanBiotech, Aidenbach, Germany). PBMC were extracted from the intermediate phase, washed in phosphate buffered saline (PBS) three times (4°C, 500 x g, 10 min) and counted with trypan blue solution (VWR Life Science, Darmstadt, Germany). Cells were either used the same day or stored in RPMI 1640 medium (PanBiotech, Aidenbach, Germany), supplemented with 1% penicillin/streptomycin and 10% heat-inactivated fetal bovine serum (both Biochrom, part of Merck, Darmstadt, Germany) overnight at 4°C, until further processing. Some horses’ cells were tested in multiple assays. No experimental animals were used in this study. Collection of blood was permitted by the local authority, Regierung von Oberbayern (Permit number: ROB-55.2Vet-2532.Vet_03-17-88).

### Magnetic Activated Cell Sorting (MACS) of CD4^+^ T Cells Using LS Columns

1 x 10^8^ PBMC were washed (4°C, 500 x g, 10 min) and then resuspended in 10 ml MACS buffer (phosphate-buffered saline (pH 7.2), supplemented with 2 mM EDTA (AppliChem, Darmstadt, Germany) and 0.5% bovine serum albumin (Serva, Heidelberg, Germany)). After another washing step, cells were incubated at 4°C with 1 ml mouse anti horse CD4 antibody (clone MCA 1078, Biorad, Feldkirchen, Germany, 1:2000) per 1x10^7^ cells. After 20 minutes, 5 ml MACS buffer were added for washing (4°C, 500 x g, 10 min). Cells were resuspended with 80 µl buffer per 10^7^ cells before adding 20 µl anti-mouse IgG1 microbeads (Miltenyi Biotec, Bergisch Gladbach, Germany) per 10^7^ cells. After incubation for 15 minutes at 4°C, another washing step (4°C, 300 x g, 10 min) was performed and cells were resuspended in 500 µl MACS buffer for cell sorting. Magnetic separation was performed using LS columns (Miltenyi Biotec, Bergisch Gladbach, Germany). Magnetically labelled CD4^+^ T cells were retained in the magnetic field, while unlabeled CD4^-^ cells were washed through the columns by three washing steps (3 x 3 ml MACS buffer). The CD4^+^ T cell fraction was eluted by removing the column from the magnetic field and rinsing with 5 ml MACS buffer. Purity of CD4^+^ fraction was tested *via* flow cytometry, by staining with 30 µl mouse anti horse CD4 FITC labelled antibody (clone MCA1078F, Biorad, Feldkirchen, Germany, 1:10) and 30 µl mouse anti horse CD8 FITC labelled antibody (clone MCA1080F, Biorad, Feldkirchen, Germany, 1:10) (**Supplementary Figure 1**, ≥ 96% purity of the CD4^+^ fraction).

### Cell Proliferation Assay

PBMC, CD4^+^ and CD4^-^ cells of controls and ERU cases were stimulated with three different mitogens to assess their proliferative response. 1 x 10^5^; cells in 200 µl RPMI 1640 medium (PanBiotech, Aidenbach, Germany) supplemented with 1% penicillin/streptomycin and 10% heat-inactivated fetal bovine serum (both Biochrom, part of Merck, Darmstadt, Germany) were seeded per well in 96-well plates. Triplicates (technical replicates) of the cells were either stimulated with pokeweed mitogen (PWM; 5 μg/mL), concanavalin A (ConA; 5 μg/mL) or phytohemagglutinin (PHA-L; 5 μg/mL) (all three Merck, Darmstadt, Germany). One triplicate of each cell type was left unstimulated as a medium control. After incubating for 32 hours (37°C, 5% CO_2_), H³-thymidine (Perkin Elmer, Germany) was added to each well (1 µCi/well) for radioactive labelling. After another 16 hours of incubation (37°C, 5% CO_2_), the cells were harvested onto a glass fiber filter and the H³-thymidine incorporation was quantified as radioactivity by liquid scintillation in a beta counter (MicroBeta, Perkin Elmer, Germany). Counts per minute were measured for each well and means of triplicates were calculated. To determine the proliferation rate, the mean value of the stimulated cells was divided by the mean value of the medium control, separately for each mitogen. No significant differences in the proliferation capacity of PBMC, CD4^+^ T cells and CD4^-^ cells of ERU cases compared to controls upon polyclonal stimulation were detected (**Supplementary Figures 2A–C**).

### Measurement of Equine Cell Metabolism Through Seahorse XFe Analyzer

Metabolic features of equine PBMC, CD4^+^ T cells and CD4^-^ cells were measured by characterizing oxygen consumption rates (OCR), extracellular acidification rates (ECAR) and glycolytic rates (glycoPER) of respective cells using a Seahorse XFe Analyzer (Agilent Technologies, Waldbronn, Germany). Following the manufacturer’s instructions, 24-well plates were coated with 52 µl Poly-D-Lysin (Merck, Darmstadt, Germany) one day prior to the assay. 8 x 10^5^ cells were seeded per well, suspended in 200 µl XF medium (Seahorse XF RPMI Medium (pH 7.4), Agilent Technologies, Waldbronn, Germany), which was supplemented with 100 mM Pyruvate, 2.5 mM Glucose and 200 mM L-Glutamine (all three Merck, Darmstadt, Germany) prior. Experiments were performed with at least two technical replicates per horse. Four wells on each 24-well plate were filled with medium and served as background correction. To ensure an evenly distributed cell layer on the bottom of each well, the plate was centrifuged for 1 minute at 2000 rpm with low acceleration and low break. All wells of the 24-well plate were filled up with XF medium to reach a total volume of 500 µl. The plate was put into a CO_2_-free incubator for 20 minutes before starting the experiment. The calibration plate was also prepared as described in the user guides of the manufacturer for each assay. OCR and ECAR rates were determined using the mito stress test (Agilent Technologies, Waldbronn, Germany). GlycoPER rates were measured with glycolytic rate assay kits (Agilent Technologies, Waldbronn, Germany). Data analysis and interpretation was done using WAVE 2.6 software according to the manufacturer’s manual (both Agilent Technologies, Waldbronn, Germany). Ratios were determined after normalizing each measurement against the baseline mean. To perform statistical analysis, data from three time-points per measurement were used.

### Western Blot

CD4^+^ T cells were lysed in lysis buffer (9 M Urea, 2 M Thiourea, 65 mM Dithioerythritol, 4% CHAPS) and protein concentration was determined *via* Bradford assay. A 12% gel was loaded with 5 µg protein/slot. Three slots were functioning as blanks. Serva Triple Color Protein Standard III (Serva Electrophoresis, Heidelberg, Germany) was used as marker. Proteins were separated by SDS-PAGE and blotted semidry onto 8.5 × 6 cm PVDF membranes (GE Healthcare, Freiburg, Germany). To prevent unspecific binding, the membrane was blocked with 4% BSA for one hour at room temperature. After three washing steps with phosphate buffered saline supplemented with 0.05% Tween (PBS-T) at room temperature for 10 minutes, it was incubated with the primary antibodies mouse anti ATP5β (Santa Cruz, Heidelberg, Germany, 1:100) and anti GAPDH (Merck, Darmstadt, 1:500) overnight at 4°C. This was followed by another three washing steps in PBS-T and subsequent incubation with POD-conjugated goat anti mouse IgG (Merck, Darmstadt, Germany, 1:5000) as secondary antibody for one hour at room temperature. After 1 hour, 6 washing steps with PBS-T were performed before re-incubation with primary antibody rabbit anti β-actin (Cell Signaling, Frankfurt am Main, Germany, 1:40000) for 3 hours at room temperature. After washing the membrane 3 x 10 minutes with PBS-T, it was incubated with POD-conjugated goat anti rabbit IgG (Life Technologies, Darmstadt, Germany, 1:10000) for one hour at room temperature. Afterwards, the membrane was washed 6 x 10 minutes with PBS-T before detection of the signals by enhanced chemiluminescence (Amersham Imager680, Analysis2.0, GE Healthcare, Freiburg, Germany). The signal intensity of ATP5β was normalized to β-actin for each horse before statistical evaluation of the differences in signal intensity between controls and ERU cases.

### Detection of Mitochondria *via* Immunocytochemical Staining

2 x 10^5^ CD4^+^ T cells of ERU cases and controls were stained with 100 µl prewarmed (37°C) MitoTracker Deep Red (ThermoFisher Scientific, Ulm, Germany, 200 nM) at 37°C and 5% CO_2_ for 30 minutes. 100 µl prewarmed (37°C) PBS were added and cells were washed (room temperature, 810 x g, 1 min), resuspended with 200 µl prewarmed PBS and washed again (room temperature, 810 x g, 1 min) before fixation in prewarmed PBS supplemented with 1% paraform aldehyde (PFA; Merck, Darmstadt, Germany) for 20 min at 37°C, 5% CO_2_. Cell nuclei were stained with 4’, 6-Diamidin-2-phenylindol (DAPI; Invitrogen, Karlsruhe, Germany, 1:100). 1 x 10^5^ cells were transferred to microscope slides and centrifuged (300 x g, 10 min) before coverslips were applied using fluoromount medium (Serva, Heidelberg, Germany). Visualization was performed using a Leica Dmi8 microscope with associated LAS-X-software, version 3.4.2 (both Leica, Wetzlar, Germany).

### Statistical Analysis

Kolmogorov-Smirnov (KS) test was used for determination of Gaussian distribution. If KS test was significant (p < 0.05; no normal distribution), Mann-Whitney test was used for statistical analysis. If KS test was not significant (p > 0.05; normal distribution), statistics were performed using student’s t-test. In both tests, statistical probabilities were considered not significant (ns) at p > 0.05 and significant at p ≤ 0.05. Significances are indicated by asterisks with * p ≤ 0.05, ** p ≤ 0.01 and *** p ≤ 0.001. OriginPro2020 software, version 9.7 (Additive, Friedrichsdorf, Germany) was used for statistical analysis and graph creation.

## Results

### PBMC and CD4^+^ T Cells Display Increased Basal Oxygen Consumption Rate (OCR) in ERU

We analyzed metabolic features of PBMC, CD4^+^ T cells and CD4^-^ cells of ERU cases and controls with a Seahorse XFe analyzer, which detects concentrations of oxygen and protons within the cell-surrounding medium. Therefore, higher oxygen consumption rates positively correlate to higher mitochondrial activity and respective oxidative phosphorylation (OXPHOS). By blocking mitochondrial respiration with rotenone/antimycin A (Rot/AA), the remaining OCR resembles the non-mitochondrial oxygen consumption, which is likely caused by the activity of oxidoreductases and other enzymes and which remains as background in all other measurements addressed in the following. Oligomycin and FCCP were used to access and influence only parts of the oxidative chain: Oligomycin inhibits the ATP synthase (complex V) of the respiratory chain reaction, which means that the decrease in OCR following oligomycin injection resembles the oxygen level used by ATP-linked respiration. Carbonyl cyanide-4 (trifluoromethoxy) phenylhydrazone (FCCP) is used as an uncoupling substance for mitochondrial respiration, thus the level of OCR after injection of FCCP shows the maximum respiration capacity of the cells.

**Figure 1:**
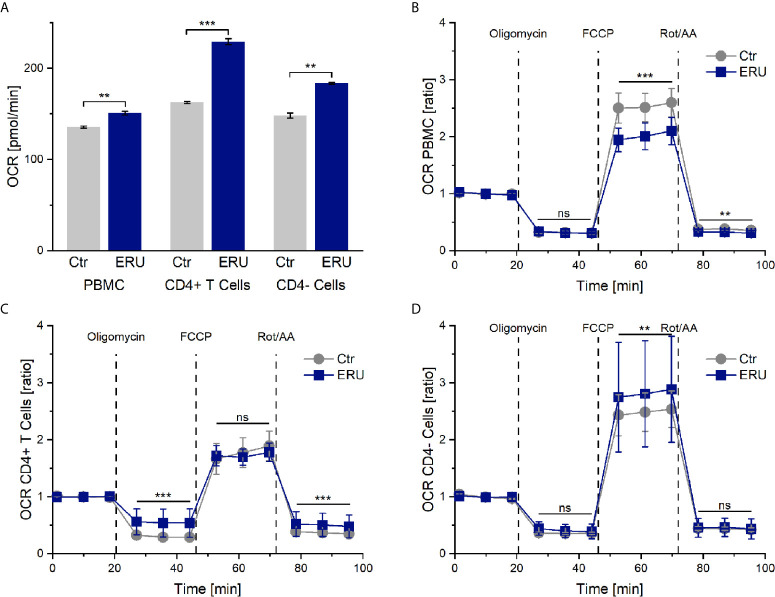
Oxygen consumption rates (OCR) and OCR ratios by mitochondrial metabolism of PBMC, CD4^+^ T cells and CD4^-^ cells of healthy and ERU cases. Controls (n=16) are presented as grey lines whereas ERU cases (n=9) are illustrated as blue lines. OCR is shown by graph **(A)** and OCR ratios are illustrated by graphs **(B–D)**. First three measurements (dots) show basal respiration, followed by injections of oligomycin (after the third measurement point), FCCP (after the sixth measurement point) and Rot/AA (after the ninth measurement point). Data are shown as mean ± SEM. **(A)** Significantly higher basal oxygen consumption rates of equine PBMC (**p ≤ 0.01), CD4^+^ T cells (***p ≤ 0.001) and CD4^-^ cells (**p ≤ 0.01) of ERU cases compared to controls. **(B)** After normalization against the baseline mean, PBMC of ERU cases showed significantly lower OCR ratio after FCCP (***p ≤ 0.001) and Rot/AA (**p ≤ 0.01) injection compared to controls. **(C)** CD4^+^ T cells of ERU cases showed significantly higher OCR ratio (***p ≤ 0.001) when injected with oligomycin and Rot/AA. **(D)** CD4^-^ cells of horses with ERU had significantly increased OCR ratio (**p ≤ 0.01) after FCCP injection. No significant difference is represented by ns (ns p > 0.05).

PBMC and CD4^-^ cells of ERU cases consumed significantly more oxygen (** p ≤ 0.01) in basal state through their mitochondria, represented through a higher OCR level compared to controls. Likewise, CD4^+^ T cells of ERU cases showed significantly increased basal oxidative phosphorylation (*** p ≤ 0.001) compared to control CD4^+^ T cells (**Figure 1A**). Furthermore, all three tested subsets of immune cells showed differing OCRs between ERU cases and controls after injection of the reagents oligomycin, FCCP and Rot/AA (data not shown). To ensure that these differing reactions to the injected reagents were independent of the different basal oxygen consumption rates between controls and ERU cases, we then normalized all measurements against the baseline mean and compared respective OCR ratios as reaction to the injected substances between the two tested groups.

### Maximum Respiration Capacity and Non-Mitochondrial Oxygen Consumption Is Lower in PBMC of ERU Cases

After adding oligomycin, no significant difference in the ATP-linked respiration was measured between PBMC of healthy and diseased groups (ns p > 0.05). However, PBMC of ERU cases had significantly decreased maximum respiration capacity (*** p ≤ 0.001) after uncoupling the mitochondria with FCCP. Non-mitochondrial oxygen consumption was also lower in PBMC of ERU cases (** p ≤ 0.01) compared to controls (**Figure 1B**).

### CD4^+^ T Cells of ERU Horses Display Decreased ATP-Linked Respiration but Similar Maximum Respiration Capacity Compared to Controls

OCR ratios of CD4^+^ T cells of ERU cases were significantly higher (*** p ≤ 0.001) compared to controls after oligomycin and Rot/AA injection. These results point to a significantly decreased ATP-linked respiration and to an increased non-mitochondrial oxygen consumption in ERU CD4^+^ T cells. CD4^+^ T cells of controls and ERU cases had similar maximum respiration capacities (ns p > 0.05) after uncoupling the mitochondria with FCCP (**Figure 1C**). In contrast, the maximum respiration capacity of CD4^-^ cells of ERU cases was significantly increased (** p ≤ 0.01) compared to controls (**Figure 1D**).

### The Extracellular Acidification Rate (ECAR) Is Basal Higher in PBMC and CD4^-^ Cells of ERU Cases

**Figure 2:**
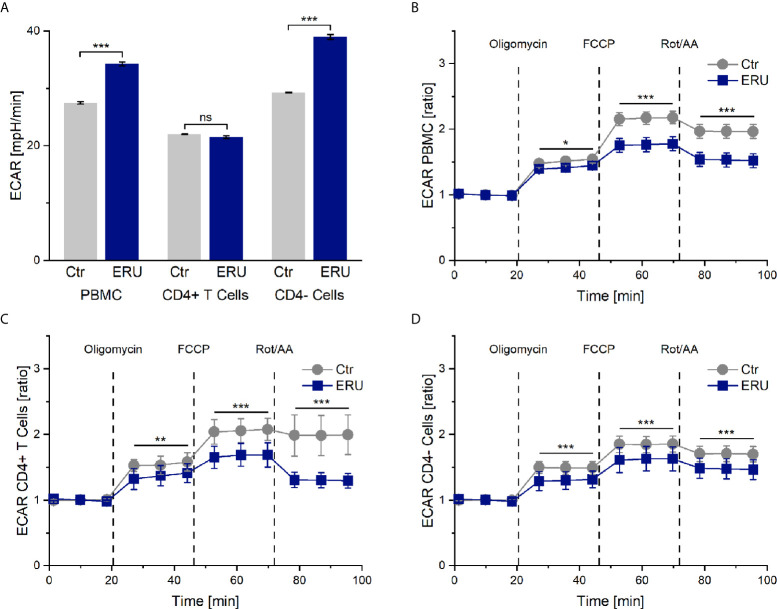
Extracellular acidification rate (ECAR) of PBMC, CD4^+^ T cells and CD4^-^ cells of healthy and ERU cases. Graphs display the extracellular acidification rate (ECAR) of cells of healthy (grey, n=14) and ERU diseased cases (blue, n=8). Graph **(A)** shows extracellular acidification rates, graphs **(B–D)** show ECAR ratios of healthy and ERU cases. First three measurements (dots) show basal respiration, followed by injections of oligomycin (after the third measurement point), FCCP (after the sixth measurement point) and Rot/AA (after the ninth measurement point). Data are shown as mean ± SEM. **(A)** PBMC and more specifically CD4^-^ cells of ERU cases showed significantly increased basal extracellular acidification (***p ≤ 0.001) compared to controls, whereas no significant difference (ns p > 0.05) between the basal extracellular acidification rate of CD4^+^ T cells of healthy and diseased horses was detectable. **(B)** ECAR ratio of PBMC of ERU cases decreased significantly after injection of the reagents of the mito stress test. **(C)** CD4^+^ T cells had a significantly lower ECAR ratio than controls after injection of oligomycin, FCCP and Rot/AA. **(D)** ECAR ratio of CD4^-^ cells was significantly lower in ERU cases than in controls after injection of the mito stress test reagents. Statistical significances are represented by asterisks (*p ≤ 0.05; **p ≤ 0.01; ***p ≤ 0.001).

Simultaneously to the measurement of the oxygen consumption rate in the mito stress test assay, the seahorse analyzer measures the pH change of the cell culture medium and provides this information as extracellular acidification rate (ECAR). ECAR mainly resembles the glycolytic activity of the cells, in the course of which lactate is produced and excreted. However, the extracellular acidification rate may also be influenced to a smaller amount by respiration linked acidification. This means that CO_2_, produced in the cause of respiration, is forming a week acid (H_2_CO_3_) in the cell-surrounding medium, which then dissociates into a proton and hydrogen carbonate. PBMC of ERU cases had a significantly higher basal ECAR rates (*** p ≤ 0.001) than control PBMC. This only applied to the CD4^-^ cells of ERU cases, whereas CD4^+^ T cells of controls and ERU cases had no significant difference (ns p > 0.05) in the basal extracellular acidification rate (**Figure 2A**). To further analyze the effects of oligomycin, FCCP and Rot/AA unaffected by differing baseline values of ERU cases and controls, all following measurements were then normalized to the baseline mean value.

### ECAR of Tested Cell Subsets of ERU Cases Decreases After Blocking and Uncoupling the Respiratory Chain

PBMC, CD4^+^ T cells and CD4^-^ cells of horses with ERU had lower extracellular acidification rates than controls after inhibiting the ATP synthase with oligomycin (PBMC * p ≤ 0.05; **Figure 2B**. CD4^+^ T cells ** p ≤ 0.01; **Figure 2C**. CD4^-^ cells *** p ≤ 0.001; **Figure 2D**). Furthermore, all tested cell subsets of ERU cases showed lower extracellular acidification than controls after uncoupling the oxidative chain reaction with FCCP (*** p ≤ 0.001) and after blocking the mitochondrial respiratory chain through Rot/AA (*** p ≤ 0.001; **Figures 2B–D**).

### The Basal Glycolytic Proton Efflux Rate (glycoPER) Is Increased in PBMC of ERU Cases, but Decreased in CD4^+^ T Cells of ERU Cases

**Figure 3:**
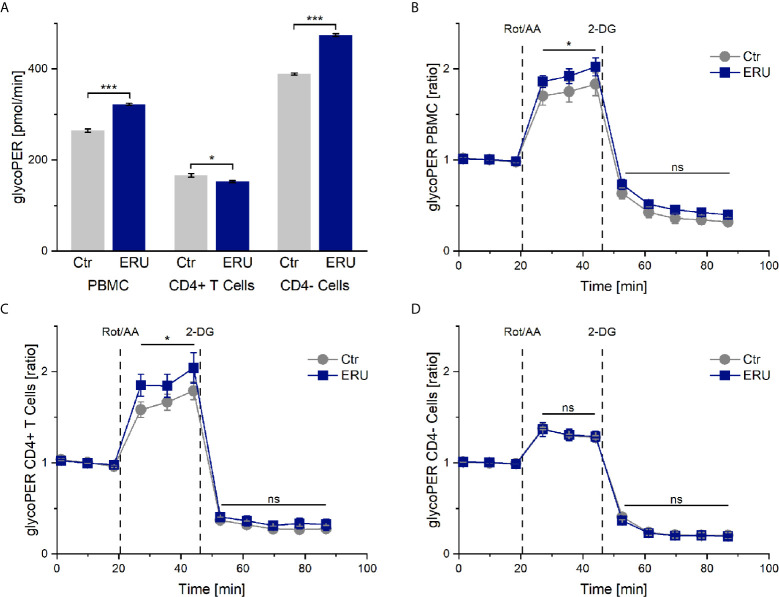
Glycolytic proton efflux (glycoPER) of PBMC, CD4^+^ T cells and CD4^-^ cells of healthy and ERU cases. Graph **(A)** shows the glycolytic proton efflux rates of controls (grey, n=8) and ERU cases (blue, n=10), graphs **(B–D)** represent glycoPER ratios, normalized against the baseline mean. First three measurements (dots) show basal respiration, followed by injections of Rot/AA (after the third measurement point) and 2-DG (after the sixth measurement point). Data are shown as mean ± SEM. **(A)** Significantly increased basal glycolysis (***p ≤ 0.001) of PBMC and more precisely CD4^-^ cells of ERU cases, whereas CD4^+^ T cells of horses with ERU showed significantly lower basal glycolysis (*p ≤ 0.05). **(B)** Significantly higher glycoPER ratio (*p ≤ 0.05) of ERU PBMC after Rot/AA injection. **(C)** Significantly increased glycoPER (*p ≤ 0.05) of CD4^+^ T cells of ERU cases after Rot/AA injection. **(D)** CD4^-^ cells showed no difference in glycolysis ratios (ns p > 0.05) after treatment with Rot/AA and 2-DG compared to controls.

Since ECAR measurement in the mito stress test represents the proton production of glycolysis indirectly, we next analyzed PBMC and CD4 sorted cells of controls and ERU cases with the glycolytic rate assay. Our goal was to determine whether resting equine immune cells also generate energy through the glycolytic pathway and whether the rate of glycolysis varied with autoimmunity. PBMC of ERU cases had significantly increased basal glycolysis (*** p ≤ 0.001) compared to controls, whereas CD4^+^ T cells of ERU cases had significantly lower basal glycolysis (* p ≤ 0.05). Furthermore, our data show that CD4^-^ cells of ERU cases caused significantly higher basal glycolytic rates (*** p ≤ 0.001) compared to controls (**Figure 3A**). As before, we normalized the measurements against the baseline mean to see whether the effects reached by injections of the two reagents of the glycolytic rate assay kit were independent of the significantly different basal glycolytic rates of ERU cases and controls.

### The Compensatory Glycolysis of PBMC and CD4^+^ T Cells Is Increased in ERU

Injection of Rot/AA inhibits the mitochondrial chain reaction and therefore mitochondrial respiration. Thus, a rise in glycoPER after Rot/AA injection resembles compensatory glycolysis. Blocking glycolysis through competitive binding of glucose hexokinase with the glucose analog 2-deoxy-D-glucose (2-DG) should lead to a decrease of glycoPER, which would confirm that the protons measured before were primarily generated through glycolysis.

Our experiments revealed a significant increase in compensatory glycolysis (* p ≤ 0.05) of PBMC of ERU cases compared to controls (**Figure 3B**). CD4 cell sorting showed that isolated CD4^+^ T cells of ERU cases caused the significantly increased levels of compensatory glycolysis (* p ≤ 0.05; **Figure 3C**), whereas CD4^-^ cells had a similar proton production as controls (ns p > 0.05; **Figure 3D**). Furthermore, there was no significant difference in glycoPER ratio (ns p > 0.05) after 2-DG injection of the tested cell subsets compared to the control group (**Figures 3B–D**). This means that the measured protons were similarly generated through glycolysis in control and ERU cases.

### CD4^+^ T Cells of ERU Cases Do Not Have Significant Difference in the Amount of Mitochondria

**Figure 4:**
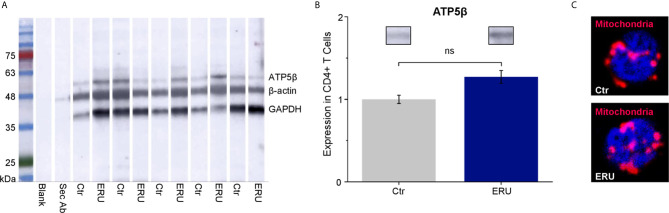
ATP5β in CD4^+^ T cells of healthy and ERU cases. **(A)** Western blot with CD4^+^ T cells of control and ERU horses (n=5 each) stained with anti ATP5β antibody, anti GAPDH antibody and anti β-actin antibody for normalization. A molecular weight marker is shown on the left, followed by a blank lane and a lane only incubated with the secondary antibody. **(B)** Statistical analysis of the volume of ATP5β lanes of control and ERU cases after normalization to the respective β-actin lane showed no significant difference (ns p > 0.05). Data are represented as mean ± SEM. The ERU bar (blue) was set accordingly to the control bar (grey), which was set as 1. **(C)** Representative immunocytochemically stained CD4^+^ T cell of a control (upper picture) and an ERU case (lower picture). Mitochondria were stained with MitoTracker Deep Red (red). Cell nuclei were counterstained with DAPI (blue). x1000 magnification.

To elucidate if the higher basal oxygen consumption rate of CD4^+^ T cells in ERU was due to more mitochondria per cell, we performed western blot analysis using the mitochondrial marker ATP5β (**Figure 4A**). CD4^+^ T cells of ERU cases and controls revealed no significant differences in ATP5β abundances, thus the total amount of mitochondria (ns p > 0.05; **Figure 4B**). Immunocytochemical staining of CD4^+^ T cells of a representative control and ERU case with the mitochondrial marker MitoTracker Deep Red illustrates this finding (**Figure 4C**).

## Discussion

Research on immunometabolism is an emerging field and new insight can help to understand pathogenesis of diseases, including autoimmune diseases, and searching for adequate therapy (27, 29, 35). To our knowledge, the link between immunometabolism and the pathogenesis of spontaneous autoimmune uveitis was not investigated so far. In this study, we therefore aimed to elucidate the metabolic phenotype and to identify metabolic alterations of leukocytes of ERU cases, which could lead to a better understanding of the autoimmune nature of the cells, the pathogenesis of uveitis and provide a basis to identify the autoreactive cells in the periphery before they can harm the eye. We showed that the metabolic phenotype of immune cells of ERU cases significantly differed in 9 of 11 tested parameters in PBMC and in 8 of 11 tested parameters in CD4^+^ T cells compared to controls, although the amount of mitochondria was unaffected by the disease. Peripheral lymphocytes are the most promising cell type to investigate ERU pathogenesis since every uveitic episode starts in the periphery, where autoaggressive T cells are activated in the spleen to migrate into the eye and destroy retinal tissue (1, 36). Caspi and colleagues further demonstrated in adoptive transfer experiments with Lewis rats that peripheral T cells specific to retinal proteins induce EAU in healthy recipient rats upon peripheral injection (37).

Since PBMC of horses with induced autoimmune uveitis had different protein expressions even in the quiescent stage of the disease compared to lymphocytes of eye-healthy horses (38), we were interested whether lymphocytes of uveitic horses also had a deviant metabolism than controls. Indeed, PBMC of ERU cases displayed a different energetic phenotype than PBMC of eye-healthy horses, namely higher basal OCR, ECAR and glycoPER rates (**Figures 1A**, **2A**, **3A**). Thus, one could conclude that the mitochondrial respiration and glycolysis pathways were both more active in PBMC of horses with ERU than in control cells. To our knowledge, this is a novel finding in the context of spontaneous uveitis, thus we do not know the functional meaning of increased basal mitochondrial respiration and glycolytic rates in uveitic pathogenesis so far. Increased basal mitochondrial respiration and glycolysis were also described in PBMC of a transgenic pig model for diabetes compared to the wild-type littermates (39). Interestingly, these findings correlate with the findings in our study concerning PBMC in recurrent autoimmune uveitis. Since the authors indicated a link between the metabolic alterations at the early stage of the disease and the inflammatory character of CD4^+^ T cells in this diabetic pig model (39), one might conclude that the increased basal mitochondrial respiration and glycolysis of PBMC of ERU cases, revealed in the present study, might also contribute to the augmented effector functions and therefore autoimmune character of these cells. A similar metabolic phenotype was seen in autoreactive lymphocytes of KBN mice, which are a spontaneous autoimmune arthritis model and were tested at an early stage of the disease (40). More precisely, CD4^+^ T cells of these KBN mice were shown to have increased basal mitochondrial and glycolytic rates compared to control mice and B cells also revealed a higher glycolytic rate than controls (40). The researchers linked the higher metabolism to the enhanced effector functions of CD4^+^ T cells and B cells of KBN mice compared to controls (40). Furthermore, they strengthened their theory by inhibiting glycolysis of KBN lymphocytes with 2-DG, which stopped the development and, when treatment was carried out early and continuous, decreased the severity of joint inflammation (40). Thus, the study managed to link the high glycolytic rates of lymphocytes in an autoimmune disease model with the disease’s severity.

PBMC are a heterogeneous cell population comprised of T cells, B cells, monocytes, dendritic cells and natural killer cells (41), thus it was unclear for us which cell types caused the observed changes in metabolism in the ERU group. By testing and comparing the metabolic reactions of CD4^+^ and CD4^-^ cells with the collective PBMC fraction, we could clarify some parameters. While discussing these results, it is important to keep in mind that the CD4^-^ cell fraction is comprised of the cell subsets in the PBMC population remaining after density gradient centrifugation and CD4 sorting, namely CD8^+^ T cells, γδ T cells, B cells, monocytes, dendritic cells and natural killer cells (41). Both CD4^+^ T cells and CD4^-^ cells caused the higher basal OCR level in PBMC of ERU cases (**Figure 1A**), whereas the highly upregulated basal glycolysis in PBMC of ERU cases was caused only by CD4^-^ cells (**Figures 2A**, **3A**). To our knowledge, we are the first to describe these differences in a direct comparison between PBMC and CD4 sorted subpopulations. Since CD4^+^ T cells are the major players in HAU, ERU and the induced uveitis models (1, 42, 43), we subsequently focused on the metabolic phenotype of this specific cell subset. We could demonstrate that CD4^+^ T cells of ERU cases utilized the oxidative phosphorylation pathway in basal state to a significantly higher amount than control cells (**Figure 1A**). Furthermore, CD4^+^ T cells of horses with ERU were engaged to a lower level in the glycolytic pathway in their basal energy state than control CD4^+^ T cells (**Figure 3A**). We therefore demonstrated that CD4^+^ T cells of ERU cases and controls had different basal metabolic phenotypes. The higher OCR level in ERU might be caused by higher mitochondrial respiration rates. To our knowledge, we are the first to describe this altered metabolic phenotype in CD4^+^ T cells of cases affected by spontaneous autoimmune uveitis. Still, these results are mostly in line with the findings of altered T cell metabolism in mouse models of other autoimmune diseases like rheumatoid arthritis or SLE (33, 40). In SLE, an autoimmune disease mainly driven by autoreactive CD4^+^ T cells and B cells (44), studies revealed an increased level of glycolysis and mitochondrial activity of CD4^+^ T cells of lupus patients and the lupus-prone mouse model (33, 44). The authors suggested that this altered T cell metabolism could cause SLE (33). With this in mind, we hypothesize that the altered T cell metabolism of ERU cases revealed in the present study might also play an important part in the pathogenesis of this disease. Functional studies are necessary to further research this interesting topic in the future, like it was recently done in a study on murine interferon regulatory factor-4 (IRF4) depleted CD4^+^ T cells of an experimental autoimmune uveitis model (EAU) (45). Here, a connection between knockout of IRF4 and lower mitochondrial respiration in basal state in CD4^+^ T cells and suppressed autoimmune uveitis could be shown (45). Unfortunately, this study did not include CD4^+^ T cells of eye-healthy control mice, thus is it uncertain whether IRF4 depletion adjusted the mitochondrial respiration level of CD4^+^ T cells of EAU cases to that of respective cells of healthy mice.

Interestingly, CD4^+^ T cells of ERU cases were able to reach higher rates in compensatory glycolysis compared to controls, when mitochondrial activity was blocked (**Figure 3C**). We hypothesize that the ability of autoreactive CD4^+^ T cells to achieve higher glycolytic rates when needed, might be due to higher expression levels of glucose transporters. In human CD4^+^ effector T cells, GLUT1 was shown to be essential, as its impairment led to decreased glucose uptake, glycolysis and effector T cell differentiation (46). Furthermore, in a mouse model of inflammatory bowel disease *in vivo*, GLUT1 deficiency led to reduced ability of effector T cells to induce inflammation (46). Autoimmune phenotypes in spontaneous and induced mouse models for SLE were ameliorated upon inhibition of glucose transport with CG-5, a glucose transport inhibitor (44). Thus, higher expression levels of GLUT1 or another glucose transporter in immune cells of uveitis cases might increase their ability to induce inflammation in the immune privileged eye, which merits further investigations in the future, as soon as antibodies for equine glucose transporters are available.

Furthermore, CD4^+^ T cells of ERU cases showed decreased ATP-linked respiration and increased non-mitochondrial oxygen consumption compared to controls, when complexes of their mitochondrial respiratory chain were blocked (**Figure 1C**). Noteworthy, these differences could not be seen when CD4^+^ T cells were tested together with CD4^-^ cells (**Figure 1B**). The reason for that might be that sorting out the CD4^+^ T cells impacts the relative proportions of the remaining cell subsets in the CD4^-^ fraction. One could speculate that subset-specific metabolic patterns could now have a stronger effect on the CD4^-^ cell metabolic curve than on the PBMC metabolic curve. Another possible explanation for this observation might be that the diverse cell subsets in the PBMC population influence each other in their metabolism, especially when some complexes of the respiratory chain or the glycolytic pathway are blocked or uncoupled. This might result in the specific metabolic curve of the PBMC group, but does not evenly represent the metabolic curves of every cell subset within this group. Taken together, our results point to altered parts of CD4^+^ T cell metabolism of horses affected by ERU, namely spending less oxygen on producing ATP through oxidative phosphorylation and in return using more oxygen for non-mitochondrial processes such as glycolysis. Our study is the first to describe these results for immune cells in autoimmune uveitis. In basic research however, effector T cells of C57BL/6 mice were shown to engage in aerobic glycolysis upon pro-inflammatory signals (27, 47, 48). We therefore hypothesize that CD4^+^ T cells of ERU cases as well rather use oxygen to engage in aerobic glycolysis than trying to compensate impaired mitochondrial function with an upregulated OXPHOS. This leap between ERU CD4^+^ T cells, obtained in the quiescent stage of the disease, and T cells in inflammatory studies is possible, because former studies demonstrated that PBMC of ERU cases are locked in an inflammatory state even in the quiescent stage of the disease. For example, PBMC of ERU cases, which were obtained in a quiescent stage of the disease, had higher lactotransferrin concentrations, which the authors linked to the presence of activated immune cells (49). ERU immune cells also demonstrated a more intense reaction to the autoantigen cellular retinaldehyde binding protein, more specifically a more directed and significantly closer movement toward this autoantigen, than control cells (1). Furthermore, the higher concentration of formin like 1 in CD4^+^ T cells of ERU cases was also linked to the activation of those cells in the quiescent stage of equine recurrent autoimmune uveitis (50).

Concerning the increased mitochondrial activity of PBMC and CD4^+^ T cells of ERU cases, another interesting aspect to investigate is if this enhanced activity influences the mass and formation of mitochondria within immune cells of ERU cases compared to controls. T cells of human SLE patients were shown to have higher mitochondrial mass than controls (51) and the increased mitochondrial mass of M2 macrophages of C57BL/6 mice with melanoma was suggested to be linked to the higher mitochondrial OXPHOS levels of these cells (52). Furthermore, mitochondrial remodeling is known to affect the metabolic status of a cell (29). For example, mitochondrial fission shifts the metabolism to aerobic glycolysis, whereas fusion processes shift the metabolism to OXPHOS (29). As a first step towards this research area, we investigated the amount of mitochondria in CD4^+^ T cells of controls and ERU cases. We could show that the mitochondrial marker protein ATP5β was expressed to same amounts (**Figures 4A, B**) and that mitochondria were located in the same cellular regions (**Figure 4C**) in health and ERU. This finding is in line with former studies concerning the equine CD4^+^ T cell proteome, where mitochondrial markers like Timm13, Tomm70, ATP5β and other ATP synthase subunits were not expressed significantly different in healthy horses and ERU cases (53). Thus, the metabolic alterations seen in ERU cases do not stem from a differing amount of mitochondria but more likely from a deviant metabolic activity. The altered metabolic phenotype of immune cells is not only investigated in diseases, but also in basic research, since the finding of functionally different cells that can be solely identified by their metabolic phenotype is intriguing. Mitochondria were shown to not only generate ATP and metabolic metabolites but also to control signaling pathways of a cell (54). By producing mitochondrial reactive oxygen species (mROS), they can influence the activation of transcription factors like HIF1α or NFAT, which ultimately resulted in functional changes of the cell, for example a differentiation into a pro-inflammatory effector T cell in mice [reviewed in (54)]. The knowledge in this field is emerging and many details of mROS generation and functioning are still not fully understood to date, whether in basic research nor in disease [reviewed in (54)]. Therefore, studies in a species like the horse and spontaneous disease models are scarce to date and build a first basis for further knowledge in this complex field.

Linking the results of the mito stress test to the amount of mitochondria and mitochondrial markers found in CD4^+^ T cells in controls and ERU cases, an intriguing fact comes to attention: Although ERU and control CD4^+^ T cells had the same amount of ATP5β, the ATP synthase (complex V) used less oxygen in ERU CD4^+^ T cells compared to controls (**Figure 1C**). Future research will be necessary to elucidate the reason for this in the equine model of autoimmune uveitis. One possible explanation might be that CD4^+^ effector T cells of ERU cases are likely to use the glycolytic pathway to a higher extend than control cells, as previously described for CD4^+^ T cells of an EAE mouse model (55). Another possible explanation could be a functional defect in complex V of the respiratory chain of ERU cells, as it was shown in immortalized human ME/CFS lymphoblasts (56).

Taken together, our findings prove a clear difference in the metabolic phenotype of peripheral CD4^+^ T cells of ERU cases and controls, which allows researchers to identify and possibly target the autoreactive cells in the periphery before they migrate into the eye and destroy retinal tissue. With an increased mitochondrial metabolism under basal conditions and the ability to switch to alternative pathways when the mitochondria are impaired and to engage in higher glycolysis when needed, CD4^+^ T cells of ERU cases are metabolically suited to lead an autoimmune attack. Overall, our results provide a basis for further investigations concerning the functional implication of the altered phenotype of autoreactive lymphocytes in the pathogenesis of autoimmune uveitis.

## Data Availability Statement

The raw data supporting the conclusions of this article will be made available by the authors to any researcher upon request.

## Ethics Statement

The animal study was reviewed and approved by Regierung von Oberbayern, Permit number: ROB-55.2Vet-2532.Vet_03-17-88.

## Author Contributions

CD conceived, designed and analyzed the experiments and supervised the project. CB, CW, AH and SH performed the experiments. CB, AH and CD analyzed the data. CB, CW, AH and CD wrote the manuscript. All authors contributed to the article and approved the submitted version.

## Funding

This work was supported by a grant from the Deutsche Forschungsgemeinschaft DFG DE 719/4-3 (to CD).

## Conflict of Interest

The authors declare that the research was conducted in the absence of any commercial or financial relationships that could be construed as a potential conflict of interest.

## Publisher’s Note

All claims expressed in this article are solely those of the authors and do not necessarily represent those of their affiliated organizations, or those of the publisher, the editors and the reviewers. Any product that may be evaluated in this article, or claim that may be made by its manufacturer, is not guaranteed or endorsed by the publisher.
