# Supplementary material for: Altered Metabolic Phenotype of Immune Cells in a Spontaneous Autoimmune Uveitis Model
Source: Front Immunol. 2021 Jul 27;12:601619. doi: 10.3389/fimmu.2021.601619 (PMC8353246; doi:10.3389/fimmu.2021.601619)
Supplement: Supplementary file 1 [file DataSheet_1.docx]

Supplementary Material

## Supplementary Figure 1


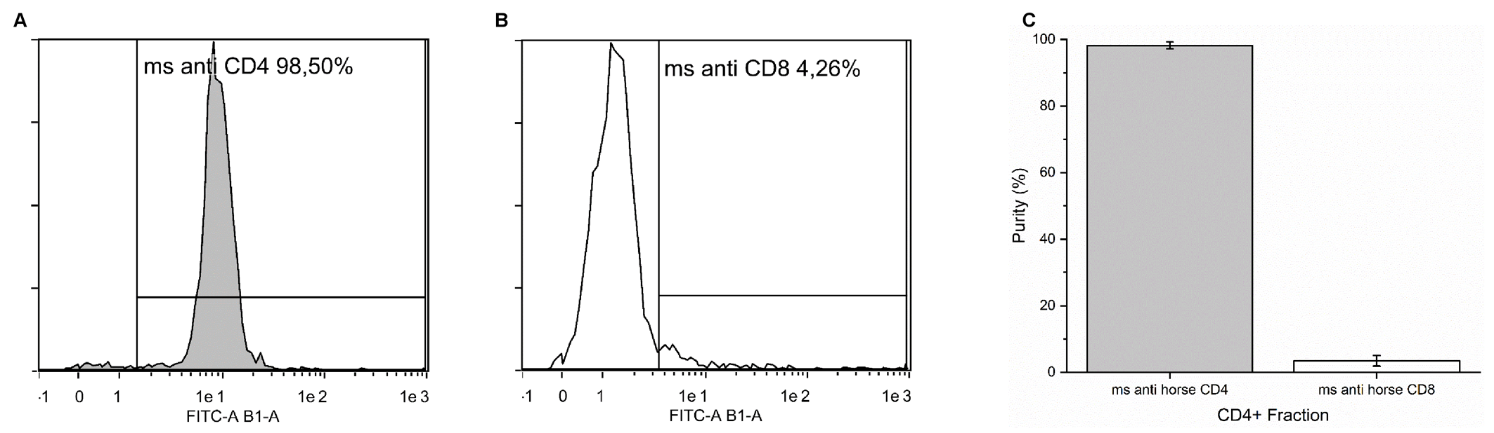


**Supplementary Figure 1:** Purity of CD4^+^ fractions after magnetic activated cell sorting (MACS). **(A)** Graph showing representative flow cytometry result of a CD4^+^ cell fraction, stained with mouse anti horse CD4 FITC antibody. **(B)** Graph showing representative flow cytometry result of a CD4^+^ cell fraction, stained with mouse anti CD8 FITC antibody. **(C)** Bar chart showing the achieved purity of tested CD4^+^ cell fractions (n=18) after sorting, stained with mouse anti horse CD4 FITC antibody (left column) and mouse anti horse CD8 FITC antibody (right column).

## Supplementary Figure 2

##
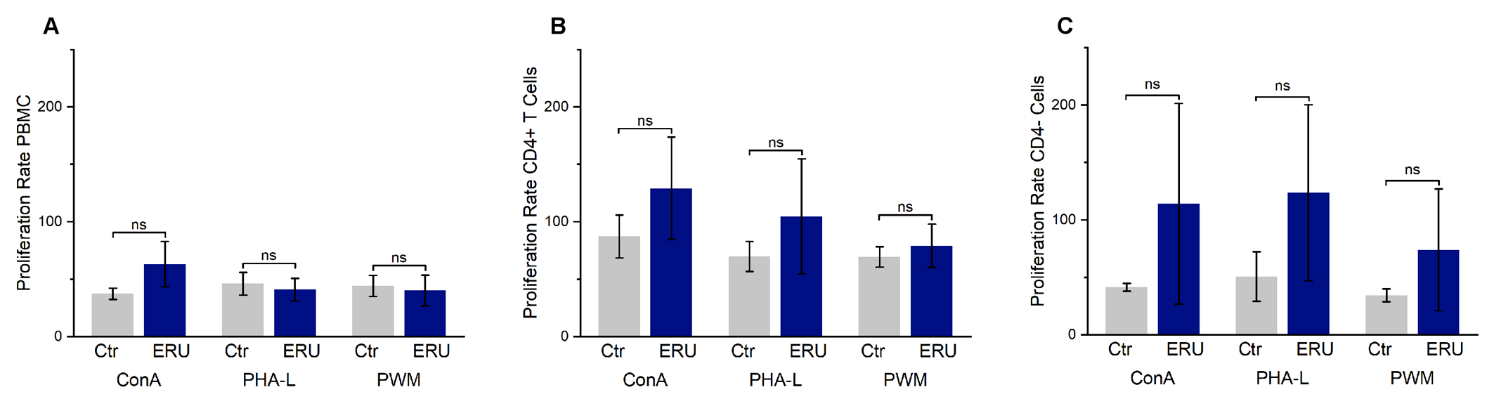


**Supplementary Figure 2:** Cell proliferation rates of PBMC, CD4^+^ T cells and CD4^-^ cells of healthy and ERU cases. Controls are represented as grey and ERU cases as blue bars. Data are shown as mean ± SEM. **(A)** No significant difference (ns p > 0.05) in the proliferation rate of PBMC of control (n=16) and ERU cases (n=6) after stimulation for 48 hours with ConA, PHA-L and PWM was measured. **(B)** CD4^+^ T cells of controls (n=3) and ERU cases (n=7) showed now significant difference (ns p > 0.05) in the proliferation rate upon ConA, PHA-L and PWM stimulation for 48 hours. **(C)** CD4^-^ cells of controls (n=3) and ERU cases (n=3) did not differ in their proliferation rate upon stimulation with ConA, PHA-L and PWM for 48 hours (ns p > 0.05).
